# Spinal Delivery of p38: TNF-alpha Inhibitors

**DOI:** 10.1371/journal.pmed.0030511

**Published:** 2006-11-28

**Authors:** Edward Tobinick

**Affiliations:** Institute for Neurological Research, Los Angeles, California, United States of America

The new study by Boyle and colleagues provides important data on basic science mechanisms involved in pain and inflammation [1]. Their data, along with that from previous studies, provides further basic scientific evidence documenting p38–TNF-alpha interactions, and suggests that spinal p38 or spinal TNF-alpha blockade may have clinical relevance [1,2]. The present study documents that p38 activation may be occurring predominantly in microglia. The present study, therefore, joins other recent work which suggests the importance of p38-glial-TNF-alpha interactions in neuroinflammation and synaptic signaling [3–6]. This increasing evidence may have clinical relevance not only to arthritis pain, but also to the pathogenesis of various forms of neuropathic pain and Alzheimer disease [1–8].

Because the present study suggests that spinal delivery may be more effective than systemic delivery when attempting to intervene in spinally-mediated inflammatory mechanisms, the authors note the potential importance of developing compounds that may bypass the blood-brain barrier. The present author speculates that the rapid and significant clinical effects noted following perispinal administration of etanercept in small pilot studies suggest that perispinal administration of p38 inhibitors may also allow these compounds to reach the spinal cord and dorsal root ganglia in therapeutically effective amounts [7,8]. It is hypothesized that this may be possible via passage through the vertebral portion of the cerebrospinal venous system, and this may explain the efficacy of perispinal etanercept in the above cited studies [7–9]. Previous studies have documented that epidural administration of large molecules may result in delivery to the endoneurial space [10]. This evidence, along with the basic scientific evidence provided by the present study of the potential clinical importance of spinal delivery, supports consideration of investigation of this novel route of administration.
